# Comparison of a new multiplex real-time PCR with the Kato Katz thick smear and copro-antigen ELISA for the detection and differentiation of *Taenia* spp. in human stools

**DOI:** 10.1371/journal.pntd.0005743

**Published:** 2017-07-07

**Authors:** Dinh Ng-Nguyen, Mark A. Stevenson, Pierre Dorny, Sarah Gabriël, Tinh Van Vo, Van-Anh Thi Nguyen, Trong Van Phan, Sze Fui Hii, Rebecca J. Traub

**Affiliations:** 1 Faculty of Veterinary and Agricultural Sciences, University of Melbourne, Parkville, Victoria, Australia; 2 Faculty of Animal Sciences and Veterinary Medicine, Tay Nguyen University, Dak Lak, Vietnam; 3 Department of Biomedical Sciences, Institute of Tropical Medicine, Antwerp, Belgium; 4 Faculty of Veterinary Medicine, Ghent University, Merelbeke, Belgium; 5 Department of Physiology, Pathology and Immunology, Pham Ngoc Thach University of Medicine, Ho Chi Minh, Vietnam; 6 Barwon Health, University Hospital Geelong, Victoria, Australia; PUCRS, BRAZIL

## Abstract

**Background:**

*Taenia solium*, the cause of neurocysticercosis (NCC), has significant socioeconomic impacts on communities in developing countries. This disease, along with taeniasis is estimated to infect 2.5 to 5 million people globally. Control of *T*. *solium* NCC necessitates accurate diagnosis and treatment of *T*. *solium* taeniasis carriers. In areas where all three species of *Taenia* tapeworms (*T*. *solium*, *Taenia saginata* and *Taenia asiatica*) occur sympatrically, conventional microscope- and copro-antigen based diagnostic methods are unable to distinguish between these three *Taenia* species. Molecular diagnostic tools have been developed to overcome this limitation; however, conventional PCR-based techniques remain unsuitable for large-scale deployment in community-based surveys. Moreover, a real-time PCR (qPCR) for the discrimination of all three species of *Taenia* in human stool does not exist. This study describes the development and validation of a new triplex Taq-Man probe-based qPCR for the detection and discrimination of all three *Taenia* human tapeworms in human stools collected from communities in the Central Highlands of Vietnam. The diagnostic characteristics of the test are compared with conventional Kato Katz (KK) thick smear and copro-antigen ELISA (cAgELISA) method utilizing fecal samples from a community based cross-sectional study. Using this new multiplex real-time PCR we provide an estimate of the true prevalence of taeniasis in the source population for the community based cross-sectional study.

**Methodology/Principal findings:**

Primers and TaqMan probes for the specific amplification of *T*. *solium*, *T*. *saginata* and *T*. *asiatica* were designed and successfully optimized to target the internal transcribed spacer I (ITS-1) gene of *T*. *solium* and the cytochrome oxidase subunit I (COX-1) gene of *T*. *saginata* and *T*. *asiatica*. The newly designed triplex qPCR (T3qPCR) was compared to KK and cAgELISA for the detection of *Taenia* eggs in stool samples collected from 342 individuals in Dak Lak province, Central Highlands of Vietnam. The overall apparent prevalence of taeniasis in Dak Lak province was 6.72% (95% confidence interval (CI) [3.94–9.50]) in which *T*. *solium* accounted for 1.17% (95% CI [0.37–3.17]), according to the T3qPCR. There was sympatric presence of *T*. *solium*, *T*. *saginata* and *T*. *asiatica*. The T3qPCR proved superior to KK and cAgELISA for the detection and differentiation of *Taenia* species in human feces. Diagnostic sensitivities of 0.94 (95% credible interval (CrI) [0.88–0.98]), 0.82 (95% CrI [0.58–0.95]) and 0.52 (95% CrI [0.07–0.94]), and diagnostic specificities of 0.98 (95% CrI [0.94–1.00]), 0.91 (95% CrI [0.85–0.96]) and 0.99 (95% CrI [0.96–1.00]) were estimated for the diagnosis of taeniasis for the T3qPCR, cAgELISA and KK thick smear in this study, respectively.

**Conclusions:**

T3qPCR is not only superior to the KK thick smear and cAgELISA in terms of diagnostic sensitivity and specificity, but it also has the advantage of discriminating between species of *Taenia* eggs in stools. Application of this newly developed T3qPCR has identified the existence of all three human *Taenia* tapeworms in Dak Lak province and proves for the first time, the existence of *T*. *asiatica* in the Central Highlands and the south of Vietnam.

## Author summary

Human taeniid tapeworms comprise three species, *Taenia solium*, *Taenia saginata* and *Taenia asiatica*. Taeniasis is a meat-borne zoonosis transmitted by the consumption of cysticerci in raw or undercooked pork for *T*. *solium* and *T*. *asiatica* (liver) and in beef for *T*. *saginata*. Accidental ingestion of *T*. *solium* eggs by humans also results in the formation of cysticerci, often in the brain, referred to as neurocysticercosis (NCC). *T*. *solium* NCC is a significant cause of morbidity and mortality owing to epilepsy in many resource-poor communities. In animals, ingestion of eggs passed by humans results in organ and/or carcass condemnation and suboptimal economic outcomes for farmers. The accurate diagnosis of *T*. *solium* tapeworm carriers is essential to monitor the success of control programs. In areas where all three species of *Taenia* tapeworms occur together, conventional diagnostic methods are unable to distinguish between the different species of *Taenia*. In this study, we develop and apply a T3qPCR capable of detecting and discriminating all three-tapeworm species in stools in a rapid and high-throughput fashion, suitable for large-scale community surveys. The newly developed T3qPCR proved superior to previously developed immunodiagnostic and conventional microscopic-based tests in terms of diagnostic sensitivity, specificity and the ability to identify and distinguish human *Taenia* species. This qPCR assay facilitated the identification of *T*. *asiatica* tapeworms in the Central Highlands of Vietnam, for the first time.

## Introduction

Humans are infected with three species of *Taenia*—*Taenia solium*, *Taenia saginata* and *Taenia asiatica* [1,2]. While *T*. *saginata* has a global distribution, *T*. *solium* is distributed mostly in developing countries of Latin America, sub-Saharan Africa and Asia, and *T*. *asiatica* is restricted to certain Asian countries [3,4]. Taeniasis is estimated to infect 2.5 to 5 million people globally [5–7]. All three-tapeworm species utilize humans as the definitive hosts (adult tapeworm) due to the ingestion of undercooked and/or raw meat or liver [1,8]. The intermediate hosts of *T*. *solium* and *T*. *asiatica* are swine whereas the intermediate hosts of *T*. *saginata* are cattle (porcine/bovine cysticercosis) [3]. Humans may also become the accidental intermediate hosts of *T*. *solium* via the ingestion of food and water contaminated with *T*. *solium* eggs, which may result in neurocysticercosis (NCC) when the cysts lodge in the central nervous system. The clinical manifestations of *T*. *solium* NCC in humans are varied, ranging from being asymptomatic to severe neurological signs and symptoms such as epilepsy, paralysis, dementia, chronic headache, blindness or even death [9–11]. The symptoms of taeniasis in humans, on the other hand, are mostly subtle and mild, and include abdominal distension, abdominal pain, digestive disorders and anal pruritus (mostly for *T*. *saginata* and *T*. *asiatica*) [12,13].

Control of NCC necessitates accurate diagnosis and treatment of *T*. *solium* taeniasis carriers to break the cycle of transmission to pigs [14]. Although all cases of taeniasis necessitate treatment, species-specific identification from an epidemiological perspective will allow better targeted control programs to be implemented aimed at interrupting the lifecycle specific to each species of *Taenia* endemic within a community and region. Several diagnostic tools for detecting taeniasis, including microscopy, copro-antigen ELISAs, sero-antibody immunoblot [15–17], and copro-DNA tests have been developed[18,19]. Of these diagnostic tools, microscopy-based examinations of fecal samples have the limitation of poor diagnostic sensitivity owing to intermittent shedding of proglottids/eggs and low specificity owing to the inability to morphologically discriminate between the eggs of *Taenia* species [20]. Although self-detection of proglottid shedding is valuable for confirmation of individual (but not community-wide) taeniasis, it is however, not always reliable especially when deep pit latrines are being utilized. Moreover, in cases of infection with *T*. *solium*, the proglottids are immobile and may be misidentified as other worms [19]. Differentiation of *T*. *saginata* from *T*. *asiatica* based on proglottid morphology is onerous and challenging [21]. The cAgELISA utilizes polyclonal antigens and more sensitive (0.85, 95% CI [0.62–0.98]) than microscopy-based methods (0.53, 95% CI [0.11–0.97]) [22], is only capable of identification to a genus level [23–25]. More recently, a cAgELISA specific to *T*. *solium* was developed [20,26]. Another drawback of the cAgELISA is that it may miss cases of *T*. *asiatica* and *T*. *saginata* in areas where other human *Taenia* tapeworms co-exist [19]. Serum antibody detecting immunoblot assays using excretory/secretory or recombinant antigens have been developed for identification of antibodies to *T*. *solium* [15,17] and *T*. *asiatica* taeniasis [16]. Immunoblot assays were highly sensitive and specific when used in *Taenia* non-endemic areas, however resulted in a high proportion of false positive results in areas endemic for *T*. *saginata* [17]. Copro-PCRs are considered superior tools for the detection of *Taenia* eggs in fecal samples owing to their high diagnostic sensitivity and the ability to distinguish *Taenia* species. Currently, the only multiplex-PCR [27,28] and PCR-RFLP [21] that is able to differentiate all human *Taenia* tapeworms is unsuited for large-scale community surveys owing to labor intensive process of restriction digest and gel electrophoresis. A better suited real-time PCR [29] has been developed but it is only capable of discriminating *T*. *solium* and *T*. *saginata*. Loop-mediated isothermal amplification (LAMP) [30] has also shown promise as a potential point-of-care diagnostic technique capable of highly sensitive detection (one copy of target gene/reaction or at least five eggs/gram of feces) and differentiation of *Taenia* spp. in stool [30], however the technology is still far from point-of-care based and is considered equivocal to PCR [19].

This study describes the development and validation of a new multiplex Taq-Man probe-based qPCR for the detection and discrimination of all three species of *Taenia* in human stools. The diagnostic characteristics of the test are compared with the test characteristics of conventional KK thick smear and the cAgELISA using hyper immune rabbit anti-*Taenia* IgG polyclonal antibody by applying all three diagnostic tests to detect *Taenia* spp. in fecal samples collected as part of a community-based cross-sectional study carried out in the Central Highlands of Vietnam.

## Materials and methods

### Ethics statement

This study was reviewed and approved by the Behavioural and Social Sciences Human Ethics Sub-committee, the University of Melbourne (reference number 1443512) and conducted under the supervision of the local Center for Public Health, Dak Lak, Vietnam. Participants positive for taeniasis on KK thick smear were treated immediately by local medical officers with praziquantel (Distocide, Shinpoong Daewoo Pharma CO.LTD) at 20 mg/kg as a single dose, whereas those positive by cELISA and qPCR were treated once results were known.

Production of polyclonal antibodies in rabbits used for cAgELISA were obtained from the Ethical Committee for animal experiments of the Institute of Tropical Medicine, Antwerp (Reference number DG012S).

### Multiplex real-time PCR

#### Primers and probes

The internal transcribed spacer I (ITS-1) gene of *T*. *solium* (GenBank accession number LC004200) was utilized to design a *T*. *solium* specific primer and Taq-Man-based hydrolysis probe for the specific amplification of *T*. *solium*. Sequences spanning the cytochrome oxidase subunit I (COX-1) gene were utilized to design a primer pair for specific amplification of *T*. *saginata* and *T*. *asiatica* concurrently, and two different species-specific Locked Nucleic Acid (LNA) probes were used for detection of *T*. *saginata*, and *T*. *asiatica*. Primers and probe of equine herpesvirus 4 (EHV4) used as an internal control were based on a previously published paper [31] (Table 1).

**Table 1:** Primers and probes for multiplex qPCR.

Target species	Oligonucleotide	GenBank accession #	Target gene	Sequence (5’ - 3’)
*T*. *solium*	Forward primer	LC004200	ITS-1	TGTTAGCAGCAGTTGTGATG
	Reverse primer			CCAACTCCACCCAGATTGA
	Probe			Cy5/CCAGCGCTG/TAO/CTGTTGACTGA/IAbRQSp
*T*. *asiatica* and *T*. *saginata*	Forward primer of *T*. *asiatica* and *T*. *saginata*	AB107234 and AB271695	COX-1	GAGTACCAACAGGAATAAAGGT
	Reverse primer of *T*. *asiatica* and *T*. *saginata*			CAACACAATACCAGTCACACC
	Probe of *T*. *asiatica*			FAM/AA+CTA+T+C+CA+CCA/IAbkFQ
	Probe of *T*. *saginata*			HEX/AA+CTA+T+T+CA+C+CA/IAbkFQ
EHV4	Forward primer	M26171	Glycoprotein gB	GATGACACTAGCGACTTCGA
	Reverse primer			CAGGGCAGAAACCATAGACA
	Probe			ROX/TTTCGCGTGCCTCCTCCAG/IAbRQSp

“+” symbols found in the probe sequences indicate the presence of a Locked Nucleic Acid Base (LNA); IAbRQSp: Iowa Black RQ-Sp; IAbkFQ: Iowa Black FQ

#### Multiplex qPCR conditions

DNA of EHV4 was spiked into T3qPCR prepared reaction mixtures as an internal reaction control. The multiplex qPCR assay was run as a 20 μl reaction containing 10 μl of GoTaq Probe qPCR Master Mix (Promega Corporation, Madison, WI, USA), 2.44 μl of H_2_O, 350 nM of each primer of *T*. *solium*, *T*. *saginata* and *T*. *asiatica*, 40 nM of each primer of EHV, 200 nM probe of *T*. *solium*, 250 nM each probe of *T*. *saginata* and *T*. *asiatica*, 100 nM probe of EHV, 1 μl of EHV4 DNA diluted 20 times and 2 μl of DNA template.

The T3qPCR amplification was carried out in a Magnetic Induction Cycler, MIC (Bio Molecular Systems). The cycling conditions consisted of an initial denaturation step at 95°C for 2 minutes, followed by 40 amplification cycles, each comprising a denaturation step at 95°C for 30 seconds and annealing at 66°C for 60 seconds. All samples were tested in duplicate with positive and negative control samples were included in each amplification assay.

#### Multiplex qPCR controls

Synthesized gBlock gene fragments of *T*. *solium*, *T*. *saginata* and *T*. *asiatica* were used as the positive controls for the T3qPCR assays. The gBlocks Gene Fragments (Integrated DNA Technologies (IDT), Coralville, USA) are double-stranded and sequence-verified DNA molecules that vouch for the fidelity of the T3qPCR assays. The gBlock fragments of *T*. *solium* and *T*. *asiatica* were synthetically combined together to an amplicon size of 235 bp in length while the *T*. *saginata* gene fragment was synthesized as an individual amplicon with a length of 140 bp (Table 2).

**Table 2:** gBlocks positive controls.

Organisms	GenBank accession number	Gene	Star-end position^1^	Amplicon length
*T*. *solium* and *T*. *asiatica*	LC004200	ITS-1	92–183	235 bp
	AB107234	COX-1	938–1077	
*T*. *saginata*	AB271695	COX-1	938–1077	140 bp

^1^ Original position in GenBank

In addition, gDNA from proglottids sourced from individuals in this study (following treatment and recovery) belonging to *T*. *asiatica* and *T*. *saginata* as well as *T*. *solium* from eggs sourced from the Centers of Disease Control and Prevention (Atlanta, GA, US) were utilized as positive controls for this study. *T*. *asiatica* and *T*. *saginata* proglottids were morphologically identified and confirmed using a previously published PCR [27] or singleplex PCR followed by DNA sequencing.

The individual qPCR reactions were optimized by subjecting individual gBlocks for each *Taenia* species to serial 10-fold dilutions ranging from 5 × 10^−7^ to 5 × 10^1^ pg/μl and then mixed together to assess the diagnostic sensitivity, specificity and efficiency of the multiplex assay. EHV4 DNA templates were also included in optimization of the multiplex qPCR. Normal melt curves and absolute quantification analyses were used to determine the positive status of individual samples. Threshold and fluorescence cut off level were set up at 0.1 and 16%, respectively, except for fluorescence cut off level of EHV at 20%, and the first cycle of qPCR amplification was ignored from the analysis. The T3qPCR results were considered negative if cycle threshold (C_t_) values were > 35. This value (5 × 10^−5^ pg/μl) was the limit of our standard curves (additional gBlocks dilutions were undetectable). Each sample was checked for fidelity/inhibition by comparing the Ct-value of the EHV4 in the sample compared to the EHV4 Ct-value in the negative control. An assay was deemed a failure when its EHV4 Ct-value was greater than two cycles different in comparison to the negative control EHV4 Ct-value.

The diagnostic specificity of the T3qPCR was confirmed utilizing DNA templates from 12 different human intestinal parasites and one canine tapeworm, including *Cryptosporidium parvum*, *Giardia duodenalis*, *Haplorchis taichui*, *Hymenolepis nana*, *Opisthorchis viverrini*, *Ancylostoma ceylanicum*, *Necator americanus*, *Trichuris trichiura*, *Ascaris lumbricoides*, *Strongyloides stercoralis*, *Blastocystis hominis*, *Clonorchis sinensis* and *Taenia hydatigena*. These parasitic DNA templates were sourced either from feces or adult worms, and confirmed using conventional PCR and DNA sequencing.

#### Multiplex qPCR confirmation

The positive results of T3qPCR were confirmed using either a conventional multiplex PCR targeting *T*. *solium*, *T*. *saginata* and *T*. *asiatica* developed by Jeon et al. (2009) [32] or a singleplex PCR for *Taenia* sp. following DNA sequencing.

The singleplex PCR utilized self-designed forward 5’-CATCATATGTTTACGGTTGG-3’ and reverse primer 5’-GACCCTAATGACATAACATAAT-3’ designed based on COX-1 gene and amplifying a gene of 350 bp. The assay comprised 2.5μl 10×CoralLoad PCR Buffer (Qiagen, Hiden, Germany), 12.5 pmol of each primer, 0.5 U of HotStar Taq DNA Polymerase, 2μl dNTPs (4 mM), 0.5 μl of MgCl_2_ (25 mM) and 1 μl of DNA, in a total volume of 25 μl. The cycling conditions consisted of an initial denaturation step at 95°C for 5 minutes, 52°C for 1 minute and 72°C for 2 minutes followed by 40 amplification cycles, each comprising a denaturation step at 95°C for 30 seconds, annealing at 52°C for 30 seconds and extension at 72°C for 30 seconds and final extension step at 72°C for 4 minutes. The singleplex PCR products were sent to Macrogen Inc. (Korea) for sequencing.

The multiplex PCR assay comprised 2.5 μl 10×CoralLoad PCR Buffer (Qiagen, Hiden, Germany), 12.5 pmol of each forward primer, 37.5 pmol reverse primer, 0.5 U of HotStar Taq DNA Polymerase, 2μl dNTPs (4 mM), 0.5 μl of MgCl_2_ (25 mM) and 1 μl of DNA, in a total volume of 25 μl. The cycling conditions consisted of an initial denaturation step at 95°C for 5 minutes, 56°C for 1 minute and 72°C for 2 minutes followed by 45 amplification cycles, each comprising a denaturation step at 95°C for 30 seconds, annealing at 56°C for 30 seconds and extension at 72°C for 30 seconds and final extension step at 72°C for 4 minutes.

#### DNA extraction of fecal samples

Samples stored in 5% potassium dichromate (w/v) were washed three times in distilled water and then subjected to DNA extraction. Approximately 250 mg washed stool was subsequently extracted using the PowerSoil DNA Isolation Kit (Mo Bio, Carlsbad, CA, USA). The protocol of extraction was in accordance with the manufacturer’s instructions, with the exception that provided beads were replaced with 1 g of Silica/Zirconia 0.5 mm beads (Daintree Scientific, Tasmania, Australia). DNA was eluted in a final volume of 100 μl of elution buffer. The quantity and quality of the extracted DNA was quantified using a NanoDrop ND-1000 spectrophotometer (Thermo Scientific, Wilmington, DE) and results were analyzed using the NanoDrop 1000 software. The DNA extracted was determined suitable when the yield was > = 2 ng/μl and the ratio of the absorbance at 260 and 280 nm and at 260 and 230 nm were > 1.70 and 2.0–2.2, respectively. The extracted DNA was stored at −20°C until use. All samples were subjected to the multiplex qPCR for the detection and differentiation of *Taenia* spp. as described earlier.

#### DNA extraction of proglottids

Proglottids were extracted using the DNeasy Blood & Tissue Kit (Qiagen, Hilden, Germany) according to the manufacturer’s instructions and eluted in 200 μl of AE Buffer.

### Coproantigen enzyme-linked-immunosorbent assay

An in-house coproantigen detection ELISA [33] with slight modifications, as described by Mwape et al. (1990) [34], was performed on the stool samples. Briefly, a mixture of an equal amount of Phosphate Buffered Saline (PBS) and stool sample stored in 10% formalin was soaked for one hour with slight shaking and centrifuged at 2000 g for 30 minutes where after supernatant was collected. The Polystyrene ELISA plates (Nunc Maxisorp, Waltham, MA, USA) were coated with capturing hyper immune rabbit anti-*Taenia* IgG polyclonal antibody diluted at 2.5 mg/ml in carbonate-bicarbonate buffer (0.06 M, pH 9.6) and incubated for 1 hour at 37°C. The plates were washed once with PBS in 0.05% Tween 20 (PBS-T20). All wells were blocked by adding PBS-T20+2% New Born Calf Serum and incubated for 1 hour at 37°C. After washing the stool supernatant of 100 ml was added to all wells and incubated for 1 hour at 37°C followed by washing five times with PBS-T20. One hundred microliter of biotinylated hyper immune rabbit IgG polyclonal antibody diluted at 2.5 mg/ml in blocking buffer was added into each well. Between two cycles of incubating for 1 hour at 37°C followed by washing five times, 100 μl of streptavidin-horseradish peroxidase (Jackson Immunoresearch Lab, West Grove, PA, USA) diluted at 1/10,000 was added. Finally, 100 ml of ortho phenylenediamine (OPD) substrate, prepared by dissolving one tablet in 6 ml of distilled water and adding 2.5 ml of hydrogen peroxide, was added to all the wells, and incubated in a dark for 15 minutes at room temperature before stopping the reaction by adding 50 ml of sulphuric acid (4 N) to each well. The plates were read using an automated spectrophotometer at 490 nm with a reference of 655 nm. To determine the test result, the optical density (OD) of each stool sample was compared with the mean of a series of eight reference *Taenia* negative stool samples plus 3 standard deviations.

### Microscopy examination

All fecal samples were microscopically examined for the presence of *Taenia* eggs using a duplicate KK thick-smear technique [35–38] and examined by trained parasitologists at Tay Nguyen University.

### Study site and sampling

**Fig 1:**
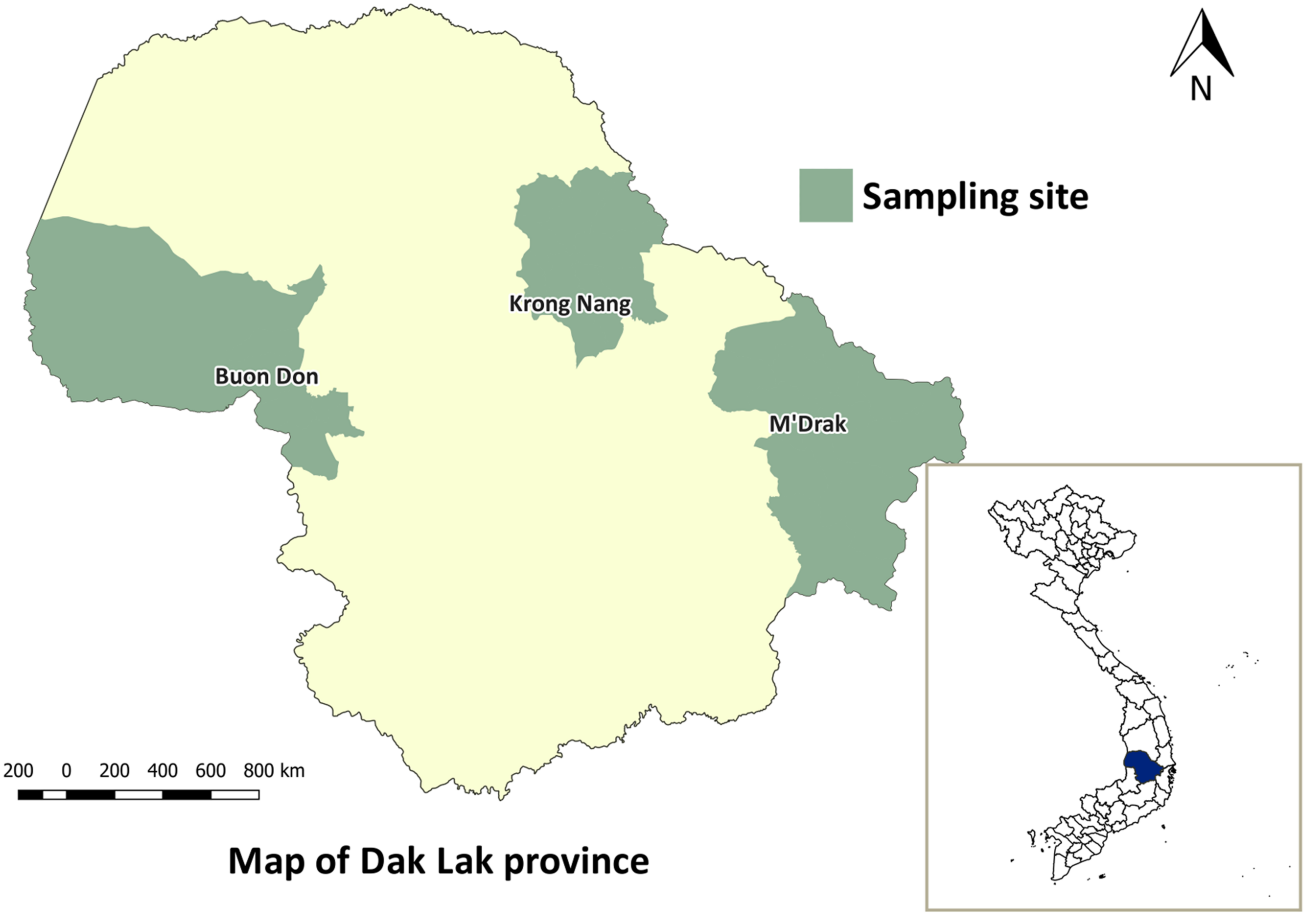
Map of study site. Map of Dak Lak province showing districts where sampling was carried out.

The fieldwork was conducted between May to October 2015 in Krong Nang, M’Drak and Buon Don districts in Dak Lak province, Vietnam (Fig 1). M’ Drak is located in the east of Dak Lak province with an average altitude of 400m to 500m above sea level and a tropical monsoon climate typical of the Vietnamese Central Coast. Krong Nang is situated to the north of Dak Lak at 800m above sea level. Buon Don, situated to the west of Dak Lak at approximately 330 m above sea level and had a hot and dry climate. Living standards in each of three districts is poor; open defecation using outdoor latrines is a common practice and livestock access to these latrine areas is, for the most part, unrestricted. Moreover, consumption of raw or undercooked blood, meat, organ tissues from domesticated and wild pigs and cattle is common. The practice of non-confinement of pigs and cattle is common in rural regions with slaughter activities commonly carried out in backyards without official meat inspection. Meat inspection is only carried out at abattoirs or slaughter-points, which serve the district level and/or clusters of large villages [32].

The objective of this study was to estimate the true prevalence of human taeniasis in each of the three study districts. Based on the previous study of Van De at al. [13], the true prevalence of human taeniasis was assumed to be 7% and there was 95% certainty that this estimate was within 5% of the true population value (i.e. the true prevalence ranged from 2% to 12%). Ignoring the tendency for taeniasis to cluster within households we estimated that a total of 100 stool samples were required. We then assumed the average number of individuals eligible for the study per household was at least three and the between household (cluster) variance in taeniasis prevalence was around 1.14 times greater than the within household (cluster) variance, returning an intra-class correlation coefficient of 0.07 [39]. Our revised sample size, accounting for the tendency of taeniasis to cluster within households, was 114 for each district and 342 for the whole province.

Within each district, three villages were selected at random. Sixty-seven households were randomly sampled from within each of the selected villages. Within each household a maximum of three individuals over the age of seven years were selected at random and asked if they would like to take part in the study. Those that consented were then asked to provide informed written consent for stool donation. Participants under the age of 18 years were recruited with assent of themselves as well as written consent from their parents or legal guardians. During the participant recruitment phase investigators informed study participants that they could withdraw from the study at any stage.

A labeled stool container was delivered to each individual. Fecal samples collected were divided into three aliquots. Approximately 5 g of each fecal sample was fixed in 5% potassium dichromate (w/v), and transported to the University of Melbourne, Australia for molecular analysis. The second and the third aliquots were stored in 5% formalin, shipped to the Institute of Tropical Medicine, Belgium, and kept at Tay Nguyen University, Vietnam, for cAgELISA testing and microscopy examination (KK), respectively.

### Statistical analysis

Statistical analyses were carried out using R (R Development Core Team 2017) [40], Microsoft Excel 2007 and Microsoft Access 2007. Cohen’s kappa test statistic (K) was used for comparison of multiplex qPCR to Kato-Katz, and multiplex qPCR to Coproantigen ELISA.

The diagnostic sensitivity and specificity of multiplex qPCR, cAgELISA and KK were estimated using a Bayesian approach. The Bayesian approach utilised prior information based on both previous literature [22] and expert opinion (Table 3). Prior information for the diagnostic sensitivity and specificity for the T3PCR was elicited from six independent molecular parasitology diagnosticians. Prior information for the sensitivity and specificity of cAgELISA and KK derived from Praet et al. (2013) [22] (Table 3). Prior information for the true prevalence of taeniasis in the three study districts was obtained from previous studies carried out in the same region [41].

**Table 3:** Parameters of prior information.

Parameter of interest		Mode	Lower limit 95%
T3qPCR*	Se	0.95	0.89
	Sp	0.99	0.94
cAgELISA	Se	0.85	0.62
	Sp	0.92	0.90
KK	Se	0.53	0.11
	SP	1.00	1.00

*Modes and lower limits for test characteristic of T3qPCR calculated as the average of the estimates elicited from each of the six experts

The model of estimation of diagnostic sensitivity and specificity for three tests applied to individuals from the same population with no gold standard test was based on Branscum et al. (2005) [42] with minor modification of the assumption that the three tests were independent [43]. The posterior distribution of diagnostic sensitivity and specificity were obtained using Markov chain Monte Carlo (MCMC) techniques. The MCMC sampler was run for 100,000 iteration and the first 1,000 ‘burn in’ samples were discarded. The point estimate and 95% credible interval (CrI) for diagnostic sensitivity and specificity were reported as the median and 2.5% and 97.5% quantiles of the posterior distribution of sensitivity and specificity. Parallel chains were run using diverse initial values to ensure that convergence was achieved to the same distribution [44]. Confirmation that the posterior estimates of the monitored parameters had converged to a stable distribution was achieved by plotting cumulative path plots for each variable [45,46] and quantified using the Raftery and Lewis convergence diagnostic [47,48].

## Results

### Optimization and validation of the multiplex qPCR

**Fig 2:**
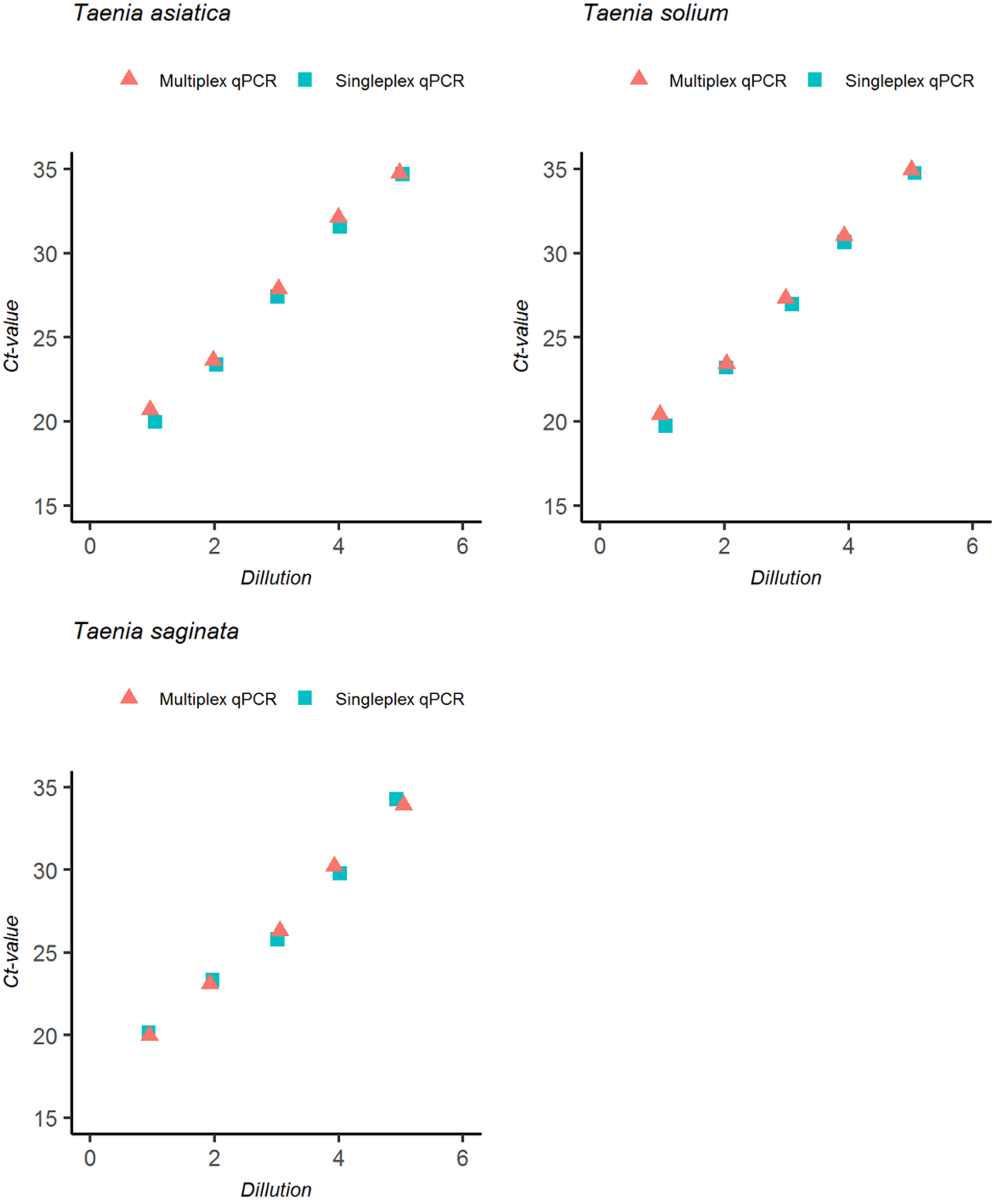
Ct value comparison of multiplex to singleplex qPCR. Assay optimization to determine effects of multiplex PCR set up on sensitivity and efficiency of PCRs compared to singleplex PCR using gBlock gene fragment (IDT Technologies) standard curve controls containing all PCR products.

Designed primers/probes for the T3qPCR were highly species-specific and failed to cross-amplify non-target parasites including other species of *Taenia*. Multiplexing the assay had no effects on the sensitivity and efficiency of the qPCR (Fig 2).

### Field study

Of 342 stool samples collected in Dak Lak province that were tested using T3qPCR and KK, 23 (6.7%, 95% CI [3.9–9.5]) and 7 (2%, 95% CI [0.9–4.4]) were positive for *Taenia* spp. For cAgELISA using a cut off of ≥ 0.2 OD, 21 (6.1%, 95% CI [3.9–9.4]) samples were classified positive for *Taenia* spp., whereas 3 (0.9%, 95% CI [0.2–2.7]) samples were classified positive for *T*. *solium* using a higher cut off of OD ≥ 0.55. The summarized test results are provided in Table 4.

**Table 4:** Results of three diagnostic tests for taeniasis.

T3qPCR					KK	cAgELISA (OD>0.2)	cAgELISA (OD> = 0.55)	Individuals
*T*. *solium*	*T*. *saginata*	*T*. *asiatica*	*T*. *solium & T*. *saginata*	*T*. *saginata & T*. *asiatica*				
0	0	0	0	0	0	0	0	306
0	1	0	0	0	1	1	0	1
0	1	0	0	0	1	0	0	5
0	0	0	0	1	1	0	0	1
0	0	0	1	0	0	1	0	1
0	0	0	0	1	0	1	0	1
0	1	0	0	0	0	1	0	2
1	0	0	0	0	0	1	1	3
0	1	0	0	0	0	0	0	6
0	0	0	0	1	0	0	0	3
0	0	0	0	0	0	1	0	13

0: Negative; 1: Positive. Raw data available in S1 Data.

Seven individuals that were positive for taeniasis by KK that were administered praziquantel, eliminated at least one adult *Taenia* worm. In all cases, the species of *Taenia* identified was consistent with that detected by the T3qPCR, including one individual that harboured co-infection with *T*. *saginata* and *T*. *asiatica*. Amplification and DNA sequencing of the individual adult tapeworms recovered from this individual showed that one of them had 99% similarity with GenBank sequence LM146668 of *T*. *asiatica* and the other to have 100% similarity with GenBank sequence JN986712 of *T*. *saginata*.

**Fig 3:**
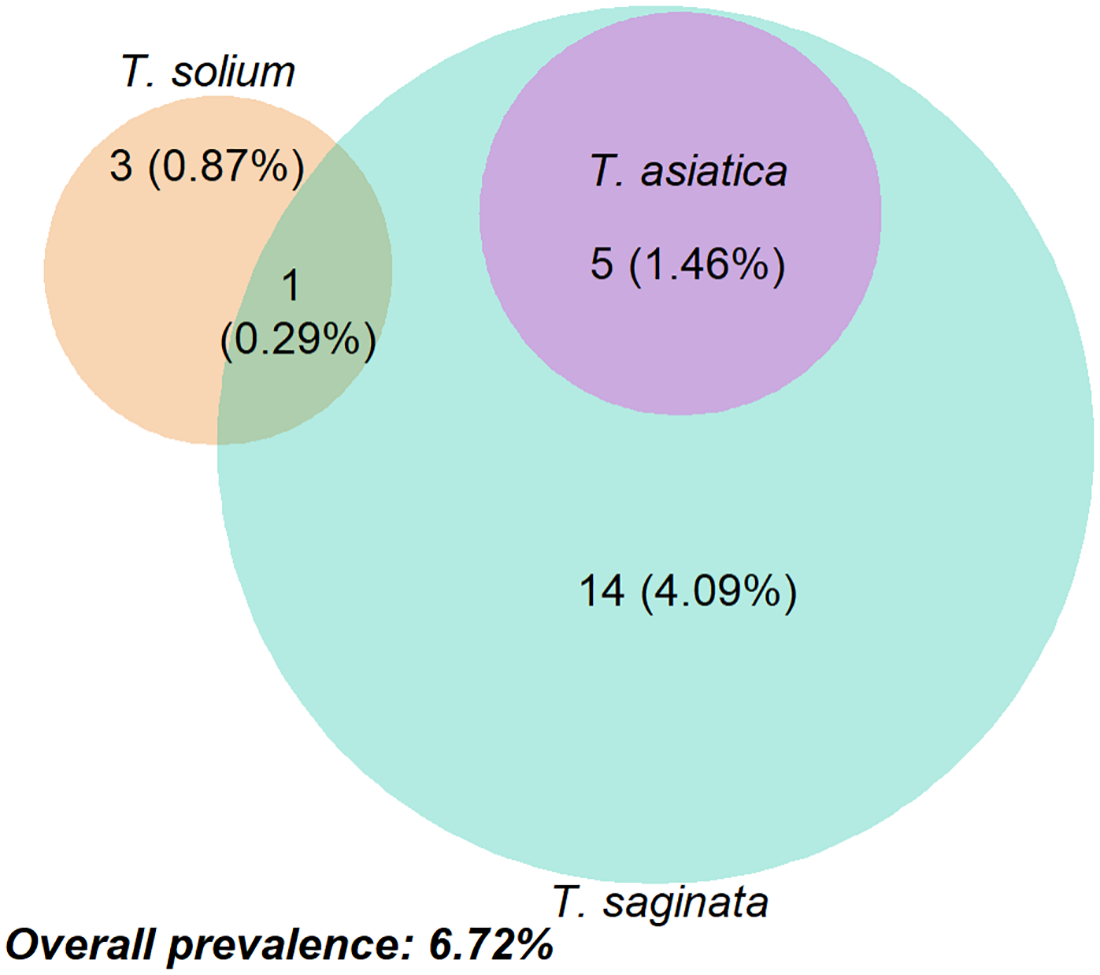
Infection status of human *Taenia* species. The venn diagram shows the proportional infection of Taenia spp. in Dak Lak province, Vietnam. Raw data available in S1 Data.

Of the 23 samples positive for *Taenia* spp. using the T3qPCR, single infections of *T*. *solium* and *T*. *saginata* were identified in 3 (0.87%) and 14 (4.09%) individuals, respectively. No cases of a single infection with *T*. *asiatica* were identified. Mixed infections of *T*. *solium* and *T*. *saginata* occurred in one individual, and of *T*. *saginata* and *T*. *asiatica* in five individuals (Fig 3).

### Comparison of T3qPCR with microscopy

The T3qPCR was more sensitive for detection of *Taenia* spp. eggs in human stools compared with KK. Twenty three of 342 individuals (6.72%, 95% CI [3.9–9.5]) were positive for *Taenia* egg using T3qPCR compared with seven of 342 individuals (2.05%, 95% CI [0.9–4.4]) positive using KK (Table 4). All the KK positive samples (7 of 342) were confirmed *T*. *saginata* and/or *T*. *asiatica* using the T3qPCR. No cases of *T*. *solium* detected by qPCR were positive by KK. Cohen’s Kappa test showed moderate agreement between the two methods (Table 5). The estimated diagnostic sensitivities of T3qPCR and KK for detecting *Taenia* spp. were 0.94 (95% CrI [0.88–0.98]) and 0.52 (95% CrI [0.07–0.94]), respectively, while the diagnostic specificity of the two tests were 0.98 (95% CrI [0.95–1.00]), and 0.99 (95% CrI [0.96–1.00]) (Table 6), respectively.

**Table 5:** The agreement statistics of T3qPCR, cAgELISA and KK for the detection of *Taenia* spp. in stool.

		T3qPCR			Kappa (95% CI)^a^	Observed agreement (%)
		Positive	Negative	Total		
cAgELISA (OD >0.2)	Positive	8	13	21	0.32 (0.21–0.43)	91.8
	Negative	15	306	321		
	Total	23	319	342		
cAgELISA (OD ≥0.55)	Positive	3	0	3	0.86 (0.75–0.96)	99.7
	Negative	1	338	339		
	Total	4	338	342		
KK	Positive	7	0	7	0.45 (0.36–0.53)	95.3
	Negative	16	319	335		
	Total	23	319	342		

^a^ Kappa agreement level: K<0.2 Poor; 0.21–0.40 Fair; 0.41–0.60 Moderate; 0.61–0.80 Good; 0.81–1.00 Very good. Raw data available in S1 Data.

**Table 6:** The estimated characteristics of three diagnostic tests for the detection of *Taenia* spp. in stool.

Test method	Parameter of interest	Median	95% credible interval (CrI)
T3qPCR	Se	0.94	0.88–0.98
	Sp	0.98	0.95–1.00
cAgELISA(OD ≥0.55)	Se	0.82	0.58–0.95
	Sp	0.91	0.85–0.96
KK	Se	0.52	0.07–0.94
	Sp	0.94	0.89–0.98

### Comparison of T3qPCR with cAgELISA

A cut off value for the cAgELISA of 0.2 was obtained by calculating the mean OD value of a series of 8-reference *Taenia* negative stool samples ± 3 SD. The cAgELISA identified 21 (6.14%) samples as positive for taeniasis however failed to detect 15 of 342 samples positive by T3qPCR. The agreement between the two diagnostic tests was fair with a Kappa value of 0.32 (95% CI [0.21–0.42]) (Table 5).

A cut off value of ≥0.55 OD on the cAgELISA was assumed to be more specific for the detection of *T*. *solium* antigens. Three (0.87%) of 342 individuals were positive for *T*. *solium* by this method in agreement with T3qPCR resulting in an apparent prevalence of 1.16% (95% CI [0.0–0.03]). The cAgELISA failed to detect any additional cases of *T*. *solium* detected by T3qPCR. There was very good agreement between the two diagnostic tests with Kappa of 0.86 (95% CI [0.75–0.96]) (Table 5). The diagnostic sensitivity and specificity of the cAgELISA was estimated to be 0.82 (95% CrI [0.58–0.95]) and 0.91 (95% CrI [0.85–0.96]), respectively (Table 6).

## Discussion

The T3qPCR developed in this study demonstrated superior ability to both the KK thick smear and cAgELISA for the specific detection and discrimination of all three taeniid tapeworms found in humans, *T*. *solium*, *T*. *asiatica* and *T*. *saginata*. The assay proved highly specific with no cross-reactivity observed for 13 different intestinal parasites as well as with each other. The cross-reactivity of the T3qPCR with *Echinococcus multilocularis* and *Echinococcus granulosus* was not evaluated as DNA of these parasites are not expected to shed in stools. The innate advantage of this T3qPCR over other diagnostic methods is its ability to detect mixed cases of *Taenia* spp. infection, a necessary tool that provides a more comprehensive understanding of the epidemiology of taeniasis and *Taenia* cysticercosis in both humans and animals in regions where all three species are sympatric [49]. For example, in communities such as those in the Central Highlands of Vietnam, where taeniasis is highly endemic, use of the T3qPCR allows the true prevalence and risk factors for *T*. *solium* taeniasis carriers to be determined. Use of these diagnostic tools to inform taeniasis control programs will not only reduce the risk of *T*. *solium* NCC in humans, but will also assist in breaking the transmission cycle to backyard and free-roaming pigs, reducing economic losses to the small-holder farmers due to carcass condemnation [50–53]. Using the Bayesian approach described in this study, the T3qPCR had an estimated sensitivity of 0.94 (95% CrI [0.88–0.98]) compared to 0.83 (95% CrI [0.57–0.98]) for a similar Taq-Man probe based multiplex qPCR described by Praet et al. (2013) [22] for the specific detection and differentiation of *T*. *solium* and *T*. *saginata*. The specificity of the T3qPCR of 0.98, 95% CrI [0.95–1.00] was equivalent (0.99, 95% CrI [0.98–0.99]) to the multiplex qPCR developed by Praet et al. (2013) [22].

Treatment and recovery followed by morphological and genetic identification of two adult tapeworms from a single individual supported the T3qPCR results to confirm the presence of *T*. *asiatica* in the Central Highlands of Vietnam for the first time, extending the known distribution of this tapeworm to southern Vietnam. This assay also confirmed the co-existence of *T*. *solium*, *T*. *saginata* and *T*. *asiatica* in the Central Highlands region. The elimination of two adult tapeworms from a single individual also confirmed the ability of the T3qPCR to detect both species of worms as opposed to infection with hybrid tapeworm. In comparison to the KK thick smear, the T3qPCR offered a significantly greater sensitivity (0.94, 95% CrI [0.88–0.99]) for the detection of *Taenia* eggs in stool samples. There were 16 *Taenia* spp. T3qPCR-positive samples not detected by KK (Table 4). The innately lower sensitivity of KK to detect eggs in feces may explain this [54]. However, the unsuitability of microscopic-based techniques for the detection of *Taenia* spp. in faeces may also be compounded by the inability of *Taenia* spp. to actively shed/lay eggs through a uterine pore like Pseudophyllidians do so [55]. As opposed to microscopy-based techniques, the T3qPCR may be in addition to any released eggs, detecting sloughed tapeworm DNA present in the stool, however this needs to be confirmed. Most significantly, KK missed all four *T*. *solium* positive individuals confirmed by T3qPCR in this community. As a result, it is our opinion that microscopic based fecal examination (KK) is neither suitable nor recommended for screening for taeniasis. Therefore, real-time PCR or cAgELISA at a higher OD cut off is recommended for screening *T*. *solium* carriers in community-based surveys in South East Asia where sympatric infection of all three human *Taenia* tapeworms is common [26,4].

The estimated sensitivity of the cAgELISA assay with an OD cut off value at 0.55 using polyclonal antibodies from hyper-immunized rabbits against excretory/secretory tapeworm antigens in this study was estimated at 0.82 (95% CrI [0.58–0.95]), twofold higher than that of KK. There was a strong agreement with the T3qPCR, in which all three cAgELISA-positive samples were confirmed as *T*. *solium* infection by T3qPCR. The sensitivity of the cAgELISA in this study was lower than that reported by Allan et al. (1996) [56] who determined test sensitivity on purgation of a subset of coproantigen-positive cases. Using a Bayesian approach, Praet et al. (2013) [22] Showed that the diagnostic sensitivity and specificity of cAgELISA was 0.85 (95% CrI [0.62–0.98]) and 0.92 (95% CrI [0.85–0.96]), respectively, similar to that estimated for this study. In total, thirteen of 21 cAgELISA OD 0.2-positive samples (Table 5) were not confirmed to be positive for *Taenia* by T3qPCR and microcopy. This suggests that the cAgELISA may cross-react with other parasites rather than *Taenia* spp [56]. cAgELISA shows excellent potential for a large scale epidemiological community-based survey in regions where sympatric *Taenia* spp. exist as it is capable of specifically identifying *T*. *solium* carriers when the cut off OD is set at ≥0.55. Therefore, choosing an appropriate OD cut off value for cAgELISA seems to be an important diagnostic consideration when applying the assay for the diagnosis of *T*. *solium* taeniasis in different communities.

In conclusion, this study describes the development and test characteristics of a new T3qPCR for the detection and differentiation of all three human *Taenia* species in stool. The absence of a diagnostic gold standard necessitated the use of a Bayesian approach to estimate diagnostic test performance. In future, the usefulness of the T3qPCR for large-scaled epidemiological studies can be confirmed using a larger sample size of individuals, ideally in a community in which the prevalence of taeniasis is higher. The assay also has the potential use in anthelmintic efficacy trials targeting individual species of tapeworms, thereby directly allowing assessment of species-specific susceptibility to drugs as well as non-chemotherapeutic interventions.

## Supporting information

S1 Data (XLSX)
